# *T*_1_ relaxometry of crossing fibres in the human brain

**DOI:** 10.1016/j.neuroimage.2016.07.037

**Published:** 2016-11-01

**Authors:** Silvia De Santis, Yaniv Assaf, Ben Jeurissen, Derek K Jones, Alard Roebroeck

**Affiliations:** a CUBRIC, School of Psychology, Cardiff University, Cardiff CF24 4HQ,UK; b Maastricht University, Maastricht, The Netherlands; c Department of Neurobiology, Faculty of Life Sciences, Tel Aviv University, Tel Aviv 69978, Israel; d iMinds-Vision Lab, Dept. of Physics, University of Antwerp, Antwerp, Belgium; e Neuroscience & Mental Health Research Institute, Cardiff University, CF10 3AT,UK

**Keywords:** Inversion recovery, DTI, Myelination, *T*_1_

## Abstract

A comprehensive tract-based characterisation of white matter should include the ability to quantify myelin and axonal attributes irrespective of the complexity of fibre organisation within the voxel. Recently, a new experimental framework that combines inversion recovery and diffusion MRI, called inversion recovery diffusion tensor imaging (IR-DTI), was introduced and applied in an animal study. IR-DTI provides the ability to assign to each unique fibre population within a voxel a specific value of the longitudinal relaxation time, *T*_1_, which is a proxy for myelin content. Here, we apply the IR-DTI approach to the human brain in vivo on 7 healthy subjects for the first time. We demonstrate that the approach is able to measure differential tract properties in crossing fibre areas, reflecting the different myelination of tracts. We also show that tract-specific *T*_1_ has less inter-subject variability compared to conventional *T*_1_ in areas of crossing fibres, suggesting increased specificity to distinct fibre populations. Finally we show in simulations that changes in myelination selectively affecting one fibre bundle in crossing fibre areas can potentially be detected earlier using IR-DTI.

## Highlights

• We apply the inversion recovery DTI approach to the human brain in vivo for the first time.
• We demonstrate that IR-DTI can measure tract-specific T1 in crossing fibres.
• IR-DTI T1 has less inter-subject variability compared to conventional T1 in crossing fibres.
• Changes in myelination affecting one fibre in crossing fibres can be detected earlier using IR-DTI.

## Introduction

White matter (WM) is organised in bundles of axons, myelinated to a varying degree, connecting specific areas of the brain. Axons tend to group into fascicles and appear to prefer to fasciculate with axons of their own type (Zipser et al., 1989); as a result, tracts consisting primarily of a homogeneous population of axons are generated (Bray et al., 1980, Kapfhammer et al., 1986, Kröger and Walter, 1991).

MRI techniques have proven to be an invaluable tool to characterise brain WM non-invasively in recent years. Rather than searching for the single MRI technique that best describes the structure of WM, there is increasing interest in multi-modal approaches, which combine different MRI techniques sensitive to distinct aspects of WM. An example is *Tractometry* (Bells et al., 2011), where the authors proposed a strategy to achieve a comprehensive multi-modal quantitative assessment of WM along specific tracts. Diffusion tensor MRI (DT-MRI) (Basser et al., 1994) allows estimation of biomarkers that reflect largely axonal properties, but are also modulated by myelin content (Beaulieu, 2002). The CHARMED approach (Assaf and Basser, 2005, Assaf et al., 2004) models water diffusion inside the axon separately from that outside the axon, providing a proxy measure of axonal density, which has been shown to correlate well with the total myelin content (De Santis et al., 2014). Q-space diffusion MRI parameters (Callaghan et al., 1990) have also been linked to the degree of myelination in recent work (Anaby et al., 2013). Myelin is believed to be an important source of contrast in $T_{2}^{*}$-weighted images from WM at high field (Lee et al., 2012, Sati et al., 2013). In addition, the longitudinal relaxation time *T*_1_ is believed to be mostly sensitive to myelin content in both WM (De Santis et al., 2014, Mottershead et al., 2003, Thiessen et al., 2013) and gray matter (Lutti et al., 2013, Stüber et al., 2014), although other factors (e.g., oedema, gliosis and axon density) affect this contrast too. MRI-based methods specific for myelin quantification have also been developed, including multi-component relaxometry (Deoni et al., 2008, MacKay et al., 1994) and quantitative magnetisation transfer imaging (Ramani et al., 2002, Sled and Pike, 2000).

With the development of more sophisticated diffusion-based approaches to reconstruct WM fibre architecture (e.g., Tournier et al., 2004, Tuch, 2004, Wedeen et al., 2008), the practice of characterising WM structure for each tract, rather than for each voxel, has become more commonplace. Since between 60 and 90% of the WM voxels contain complex fibre architecture that can no longer be described by a single WM fibre population (Jeurissen et al., 2012), it is necessary to apply techniques that are capable of resolving crossing fibres within the voxel, to provide tract-specific measures. If the aforementioned hypothesis that axonal bundles are structurally homogeneous holds, this is expected to increase biological specificity and to facilitate the detection of tract-specific properties, and their changes under learning, development and disease in conditions affecting only one population out of many crossing in the same voxel. While several methods have been proposed to assign distinct diffusion properties to distinct fibre populations, e.g. fibre-specific axonal density (Assaf and Basser, 2005, Assaf et al., 2004), orientational anisotropy (Dell’acqua et al., 2012), apparent fibre density (Raffelt et al., 2012) and axonal diameter (Zhang et al., 2011), methods developed to quantify myelin to date provide only a single (i.e., average) myelin content of the voxel, irrespective of the architectural paradigm.

Recently, a new MRI technique combining inversion recovery and diffusion, called IR-DTI, was introduced to provide fibre-specific estimates of the relaxation time *T*_1_ and of the diffusion tensor (Barazany and Assaf, 2012, De Santis et al., 2015). This technique was applied to fixed tissue of an animal model, showing the ability to provide tract-specific values of *T*_1_ in crossing areas, reflecting differential myelination properties. Here, we apply this technique in vivo to the human brain for the first time. Specifically, our aims are: 1) to prove feasibility of IR-DTI for human applications; 2) to characterise tract profiles using tract-specific values for *T*_1_; 3) to compare IR-DTI to conventional *T*_1_ measures in their ability to discriminate multiple *T*_1_s in a voxel; and 4) to compare the sensitivity to tract specific *T*_1_*changes* of IR-DTI to that of conventional single *T*_1_ maps in areas of crossing fibres.

## Methods

### Model

Conventional inversion recovery (IR) fits a single relaxation time *T*_1_ for each voxel, according to:

(1) $$S/S0=1-2\cdot \exp\;\left(-TI/T_{1}^{IR}\right)$$

If the voxel is composed of more than one *T*_1_ component, it is in principle possible to perform a multi-exponential fit on the same IR data, e.g., according to:

(2) $$S/S0=\sum_{i}f_{i}\cdot \left[1-2\cdot \exp\left(-TI/T_{1}^{i}\right)\right]$$

where *i* is the number of *T*_1_ components. However, separating two or more exponential decays with similar rates may be very difficult, because of well recognised difficulties (Touboul et al., 2005).

IR-DTI instead provides the possibility of recovering multiple relaxation times within a voxel by exploiting the orientational dependence of the diffusion signal. The IR-DTI protocol comprises several inversion recovery-prepared diffusion MRI series acquired for different inversion times (TI). The combined contrast is described by the following equation (De Santis et al., 2015):

(3) $$S/S0=\underset{i}{\sum }f_{i}\cdot \left[1-2\cdot \exp\left(-TI/T_{1}^{i}\right)\right]\cdot \exp\left(-bg^{T}D_{i}g\right)$$

The IR-DTI signal is modelled as a summation over *i* populations, each characterised by a volume fraction, *f*_*i*_, a specific diffusion tensor *D*_*i*_ and a specific $T_{1}^{i}$. In the original implementation, two populations were fitted in each voxel of the rat brain. To account for more complex fibre arrangements found in human WM, and to avoid overfitting in areas of coherent orientation, here a model selection strategy is applied to decide how many compartments should be fitted voxel-to-voxel, based on Jeurissen et al. (2012).

### Simulations

Simulations were used to test the capability of IR-DTI to disentangle multiple components in a crossing fibre voxel and to compare it with conventional IR. Eq. (3) was simulated using two different geometries: two crossing fibres, oriented along x- and y-axis, associated with *T*_1_s of 800 and 1000 ms respectively, and three crossing fibres, oriented along x-, y- and z-axis, associated with *T*_1_s of 800, 1000 and 1200 ms respectively. The fibres had identical diffusion properties (diffusion parallel to the fibre D = 1.3 * 10^ −3^ mm^2^/s), but had different volume fraction (0.4 and 0.6 in case of two fibres, 0.26, 0.33 and 0.41 in case of three fibres respectively). In addition, the angle between the first two fibres was changed in the range 30°–90°. The scheme used had the following parameters: TI = 175,250,300,350,400,450,500,585,675,750,850,1100,1500 ms, 15 non-collinear gradient orientations plus 2 unweighted scans for each TI for a total of 221 measurements, b = 1000 s/mm^2^. 100,000 noisy repetitions were generated adding noise to generate Rician-distributed data at signal-to-noise ratio (SNR) = 20 in the unweighted scan, which is a conservative estimate of the SNR achievable in vivo (see Section Data processing). Eq. (1) for two fibres, associated with *T*_1_s of 800 and 1000 ms, respectively, was simulated using the same total number of measurements (221), the same TI range (175–1500 ms, 221 equally-spaced datapoints), the same number of repetitions (i.e. 221 measurements) and the same SNR, but without diffusion weighting. IR-DTI data were fitted to Eq. (3); the orientational information was assumed to be equal to the true value in the fit, to mirror what is done in vivo (see Section Data processing). IR data were fitted to Eq. (1). To test the minimum effect size needed by IR-DTI and IR to detect a statistically significant *T*_1_ change, the same simulations were repeated for different values of the two *T*_1_s, simulating an increase of up to 10*%* in the smallest and in the largest *T*_1_, respectively. Data were then fitted to both Eqs. (3) and (1), and the difference with the original value was evaluated. The difference was compared with the variability measured in vivo for the different tracts, reported in Table 2 (see Subsection Statistical analysis).

### Data acquisition

7 healthy subjects with no history of neurological diseases participated in the study. Mean age (standard deviation) was 29 ± 6 years; 4 of the participants were males and 3 were females. The experimental procedures were approved by the ethics committee of the Faculty for Psychology and Neuroscience at Maastricht University, and were performed in accordance with the approved guidelines and the Declaration of Helsinki. Informed consent was obtained from each participant before conducting the experiments. To minimise the effects of *B*_1_ inhomogeneity, dielectric pads (Snaar et al., 2011, Teeuwisse et al., 2012) were placed between the subject's head and the coil, positioned in correspondence with temporal and occipital lobes, i.e., the brain areas most affected by such inhomogeneity due to low transmit efficiency in a volume transmit coil. The protocol comprised an IR-DTI protocol at 7T with an IR-prepared pulsed gradient spin echo (PGSE) echo planar imaging (EPI) sequence using the following parameters: TI = 200, 300, 400, 500, 700, 1000, 1500 ms, b-value 1000 s/mm^2^, 30 directions for each TI, TE = 50.8 ms, TR > 10 s, GRAPPA factor = 2. A separate HARDI scan, which is needed to recover the fibre orientations and reconstruct fibre tracts, was acquired using 60 gradient orientations, b-value 2000 s/mm^2^ and 6 b0, TE = 57.6 ms, TR = 5 s, GRAPPA factor = 3. The resolution of all diffusion scans was 2 mm isotropic and the matrix size was 96 × 96. 21 slices were acquired for IR-DTI, 45–50 (depending on the head size) for the HARDI scan in order to get full brain coverage. The slice package was centred in the corpus callosum. A high-resolution quantitative *T*_1_ scan was also acquired for each participant using an MP2RAGE sequence (Marques and Gruetter, 2013, Marques et al., 2010), at two different inversion times (TI = 900 and 2750 ms) at the resolution of 0.7 mm isotropic. The scan time for the HARDI scan was 5.5 min, while the MP2RAGE acquisition took 9.5 min; the scan time for the IR-DTI scan varied depending on the subject (due to a variable TR adjusted to the specific absorption rate constraints of each subject) and is reported in Table 1, along with the total acquisition time.

### Data processing

The SNR of the b0 image associated to the longest TI was calculated using the difference method (Murphy et al., 1993) and returned values between 25 and 30 across white matter. Data were corrected for motion and distortion and analysed using ExploreDTI (Leemans et al., 2009) and an in-house program written in Matlab (R2012B, The Mathworks). First, HARDI data were processed to obtain the fibre orientation density (FOD) using constrained spherical deconvolution (CSD) (Tournier et al., 2004). Then, up to three FOD maxima, corresponding to the main underlying fibre orientations, were extracted using a numerical optimization procedure described by Jeurissen et al. (2012). The orientational information and the volume fractions were then fed into the IR-DTI routine and kept fixed during the following steps. The routine fits a *T*_1_ value for each fibre population present in the voxel, according to Eq. (3). Hence, the fitted parameters are: (up to) three $T_{1}^{i}$, (up to) three *D*_*i*_ and a Johnson noise term (Assaf et al., 2004). The fit is performed using the *lsqnonlin* function in Matlab. The routine fits a specific longitudinal diffusion coefficient for each fibre, while the orthogonal diffusivities are derived from the longitudinal diffusion using a tortuosity model (Szafer et al., 1995). We have shown recently (De Santis et al., 2016) that the use of this tortuosity model may bias estimates of axonal density in models with both hindered and restricted diffusion compartments. However, this bias does not apply to the IR-DTI model as the images are acquired with the same diffusion time and the model does not estimate fibre density. Quantitative *T*_1_ maps, which are estimated from the two inversion times (Marques et al., 2010), were obtained directly from the scanner as output of the MP2RAGE pipeline (MP2RAGE *T*_1_); in addition, *T*_1_ maps were also computed taking the unweighted diffusion data at varying TI (IR *T*_1_) according to Eq. (1). A bi-exponential fit was also performed, according to Eq. (2).

### Statistical analysis

Due to the limited spatial resolution of the scan, the cingulum bundle (CING) and the genu of the corpus callosum (CC) effectively cross within a voxel, providing a good test-bed to demonstrate that the analysis can measure fibre specific *T*_1_ values in areas of crossing fibres. The CC and the CING were reconstructed by manually selecting waypoints for all subjects. Fibre-specific histograms of *T*_1_ were computed by assigning the $T_{1}^{i}$ values specific to IR-DTI orientations in each voxel to a specific fibre bundle: left-right were assigned to the CC, while anterior-posterior were assigned to the CING. To investigate a crossing of three fibres, the CC, corticospinal tract (CST) and superior longitudinal fasciculus (SLF) were manually reconstructed using waypoints for one selected subject. Only the lateral portion of the CC intersecting the other two tracts was considered in the analysis. Fibre-specific histograms of *T*_1_ were computed by assigning the $T_{1}^{i}$ values in each voxel to a specific fibre bundle by selecting voxel-wise the *T*_1_ associated with the orientation subtending the smallest angle to the fibre trajectory. To perform statistical analysis over all subjects and over different tracts, an automated tract segmentation approach (Yeatman et al., 2012) was chosen to reconstruct the corpus callosum, the cingulum, the thalamic radiation, the arcuate and the superior longitudinal fasciculus for each subject. The same routine was also used to extract tract profiles. The IR-DTI approach yields up to 3 *T*_1_ values at each point along every tract, and each *T*_1_ is associated with a particular orientation. To generate a tract-specific *T*_1_ profile, the algorithm selects the *T*_1_ associated with the orientation subtending the smallest angle to the fibre trajectory. Also conventional IR *T*_1_ and MP2RAGE *T*_1_ profiles were calculated by projecting the values of *T*_1_ onto the tract profile. For each tract, the standard deviation within the tract was calculated and averaged over subjects, to have an estimate of the variability of *T*_1_ in each tract. The results are reported in Table 2.

All *T*_1_ profiles were calculated for each subject and combined to find the mean profile and the associated standard deviation over all subjects.

## Results

### Simulations

**Fig. 1:**
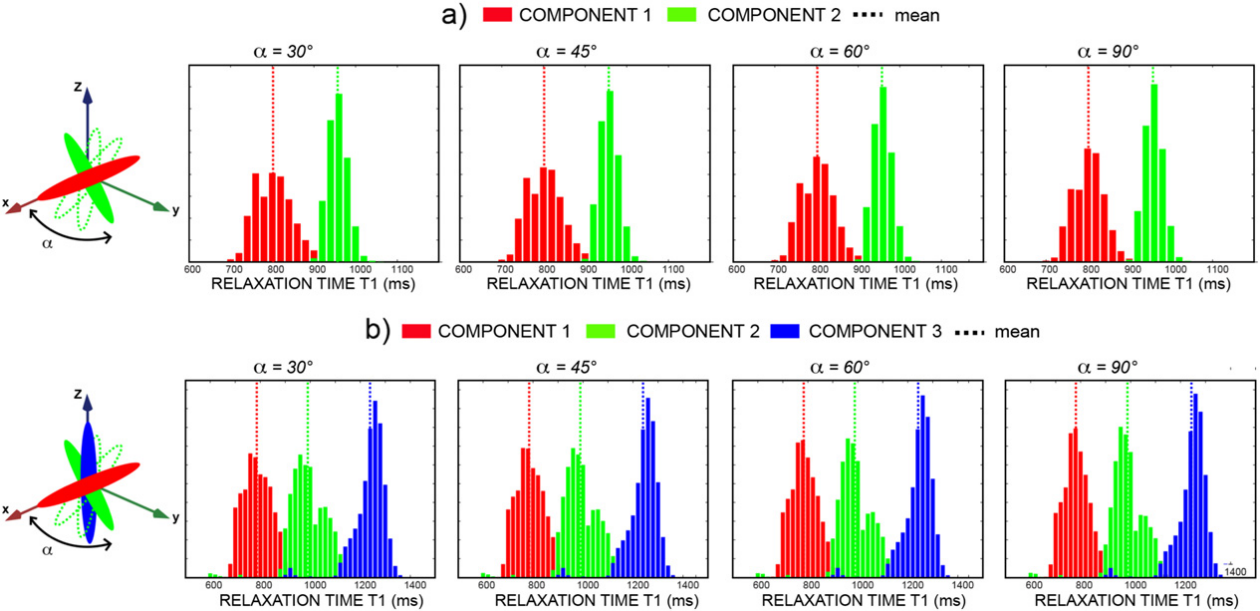
Histograms of fitted *T*_1_s in simulated data. Eq. (3) was used for double (panel a) and triple (panel b) crossing configurations at varying crossing angle between the first and the second component, as depicted in the schematic figures at the left. Dotted lines are measured mean values.

Fig. 1 shows histograms of fitted *T*_1_s to the simulated crossing fibre data using Eq. (3) for double and triple crossing, respectively, and for varying crossing angles. IR-DTI successfully recovers the distinct peaks corresponding to the different *T*_1_s even for small crossing angles.

**Fig. 2:**
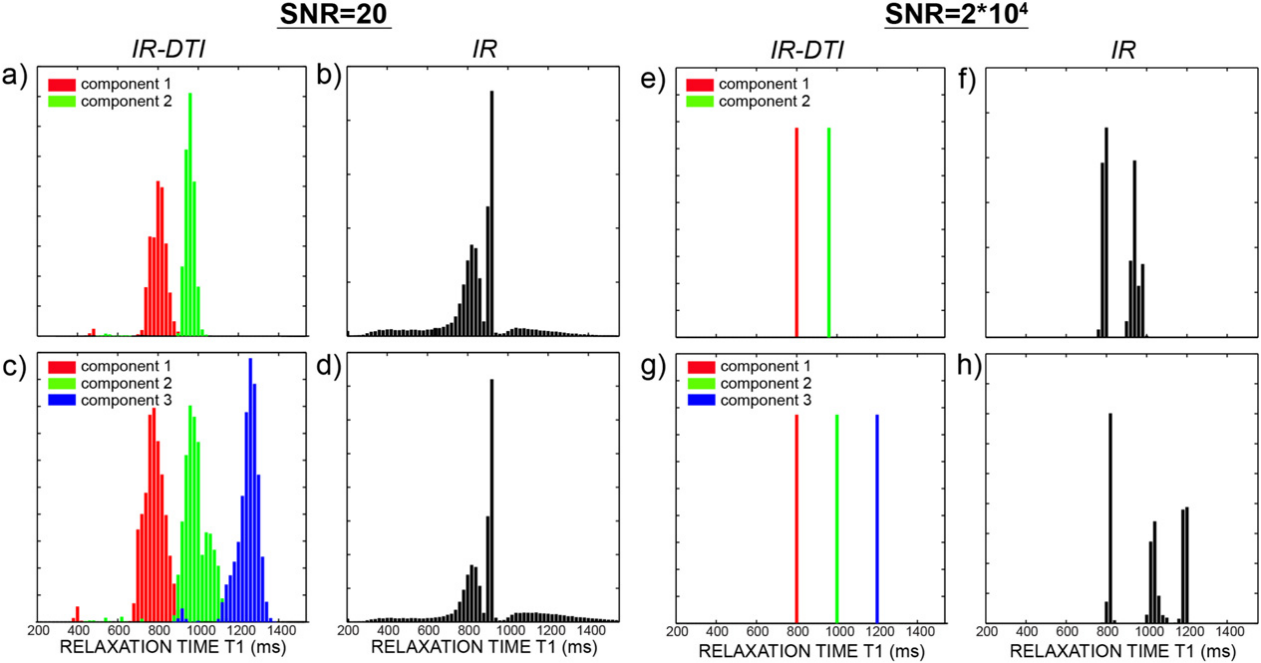
Histograms of fitted *T*_1_s in simulated data using Eq. (3) for double (a) and triple (c) crossing configurations for 90° crossing, and using Eq. (2) for two (b) and three (d) *T*_1_ components, with SNR = 20. The same is also shown for SNR = 2 * 10^4^ in plots e, f, g, h.

Fig. 2 shows the histograms of *T*_1_ for two and three fibres crossing calculated with IR-DTI and with Eq. (2), respectively, for typical SNR and for an unrealistically high SNR. The IR-DTI data are multi-variate (i.e. in the space of gradient orientations and in the space of different TI), while the IR data are univariate (in the space of TI only), but the two simulated acquisitions have the same number of datapoints. Fig. 2 shows that for typical SNR, IR-DTI can resolve fibre crossing, while a biexponential fit fails. This is likely due to the fact that when the two characteristic exponentials are close to each other, the composite becomes much harder to fit with a bi-exponential model for univariate data, and one has to go to unrealistically high SNR to resolve the different exponents.

**Fig. 3:**
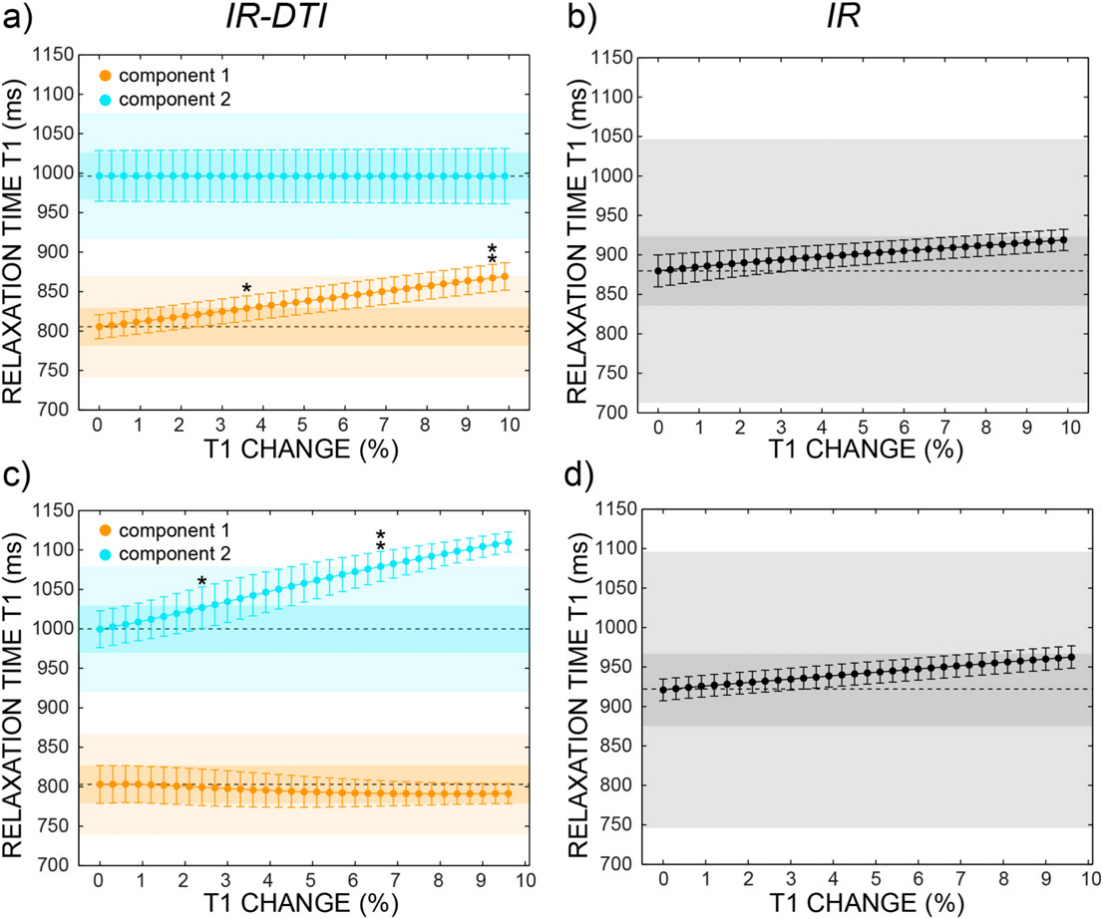
Estimated relaxation times *T*_1_ versus per cent change of true *T*_1_, reported for the two components extracted using IR-DTI (panel a and c) and for the single component extracted using IR (panel b and d) in simulated data. The results are repeated increasing the smaller *T*_1_ (panel a and b) and increasing the larger *T*_1_ (panel c and d). Error bars are standard deviations across noisy repetitions. The shaded areas represent the minimum (darker) and the maximum (lighter) variability found in vivo inside tracts. A single asterisk means that the observed change is larger than the variability measured in the tract with the smallest variability (SLF for both IR-DTI *T*_1_ and IR *T*_1_), while double asterisk means that the observed change is larger than the variability measured in the tract with the largest variability (thalamic radiation for IR-DTI *T*_1_ and CC for IR *T*_1_). Dashed lines represent initial (i.e., unchanged) values of *T*_1_.

Fig. 3 shows the minimum effect size needed to detect a change in *T*_1_ in a two fibre crossing configuration, if the change only affects one of the two fibres. The simulations are repeated for the case in which the smallest *T*_1_ is increasing (upper panels) and for the case in which the smallest *T*_1_ is increasing (lower panels). The observed change is compared with the maximum and minimum variability found in vivo (shaded areas) and reported in Table 2. IR-DTI can detect changes for effect sizes as small as 3% in tracts with low *T*_1_ variability, and needs a change of 6–9% in tracts characterised by high variability. IR, which measures the weighted average of the values between the two fibres, needs much larger effect sizes (>10%). A bi-exponential fit, according to Eq. (1), systematically fails in detecting two different values of *T*_1_ (data not shown).

### In-vivo results

**Fig. 4:**
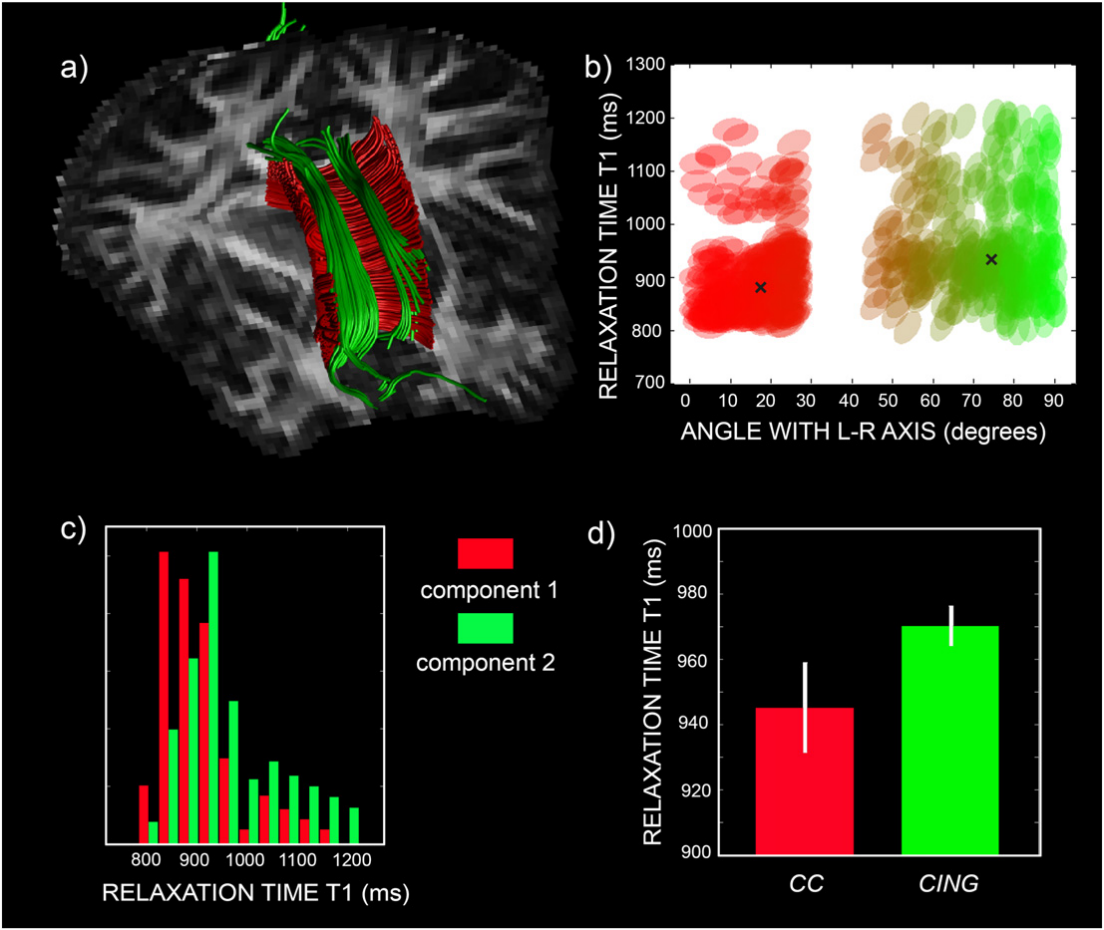
Tractography of corpus callosum (CC) and cingulum (CING) (a), scatterplot of the angle to the L-R direction versus *T*_1_ in the area of crossing of the two tracts (b) and corresponding colour-coded histogram (c). For the same two tracts, mean and standard deviation of tract-specific *T*_1_ across all subjects are reported in panel d.

Fig. 4 replicates in humans the results already obtained in rodent (De Santis et al., 2015). Fig. 4a shows the tractography of the CC and the CING in a single subject, and Fig. 4b is a scatterplot of the angle to the L-R axis versus *T*_1_ in the area of crossing of the two tracts. The two crosses represent the calculated cluster centroids. The two bundles show a clear separation on both axes, with the CING having larger *T*_1_ values than the CC. This is also shown in the histogram of Fig. 4c. In Fig. 4d, mean and standard deviation across all subjects are reported, proving that the difference in the *T*_1_ relaxation properties of the two tracts is consistent across subjects.

**Fig. 5:**
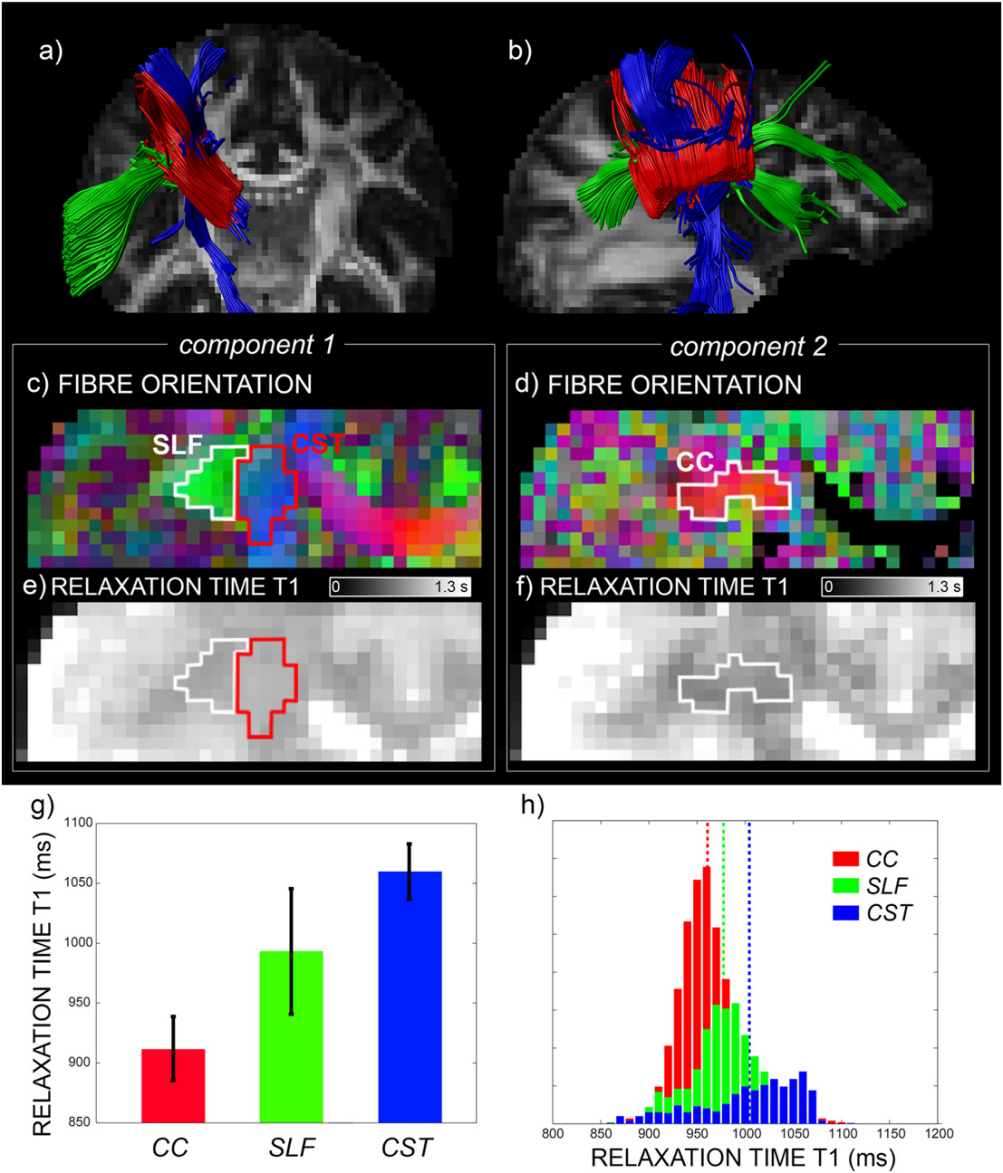
An area of crossing between the corpus callosum (CC), corticospinal tract (CST) and superior longitudinal fasciculus (SLF) for one selected subject. In panels a and b, the tractography reconstruction of the three tracts is shown. Maps of the voxelwise fibre orientations (panel c and d), along with the corresponding *T*_1_s (panel e and f) for each of the two first identified IR-DTI components, are reported for a slice comprising the three tracts. Fibre orientation is displayed using the following colour convention: red colour represents fibres passing in mediolateral orientation; green: anterior-posterior; blue: inferior-superior. In panel g, the mean and standard deviation of the relaxation time *T*_1_ is shown, calculated in the ROIs highlighted in panel c and d, corresponding to the area of crossing between the three tracts. In panel h, the histograms of *T*_1_ values across the entire tract and the corresponding average value (dotted lines) are reported for CC, CST and SLF.

Fig. 5 shows an area of triple crossing between the CC, CST and SLF. In areas likely belonging to the same fibre tract, the fitted tract-specific *T*_1_ maps are also relatively homogeneous, with adjacent tracts showing clearly distinct values of *T*_1_ (see Fig. 5e–f). This is quantified in the bar plot in the lower right panel (Fig. 5g), which shows different average *T*_1_ values for the different tracts and modest standard deviation of *T*_1_ values within the ROIs depicted in panel c and d. A two-sample *t*-test confirms that the distribution of *T*_1_ is different in the three ROIs (*p* < 0.05). To check consistency of the differences in *T*_1_ measured in the crossing area, in panel h the histograms of the tract-specific *T*_1_s are reported for the whole tracts CC, CST and SLF, reconstructed using tractography. CC is characterised by the smallest *T*_1_, while CST shows the largest *T*_1_. The values recovered in the crossing areas with IR-DTI are consistent with those calculated along the whole tract.

**Fig. 6:**
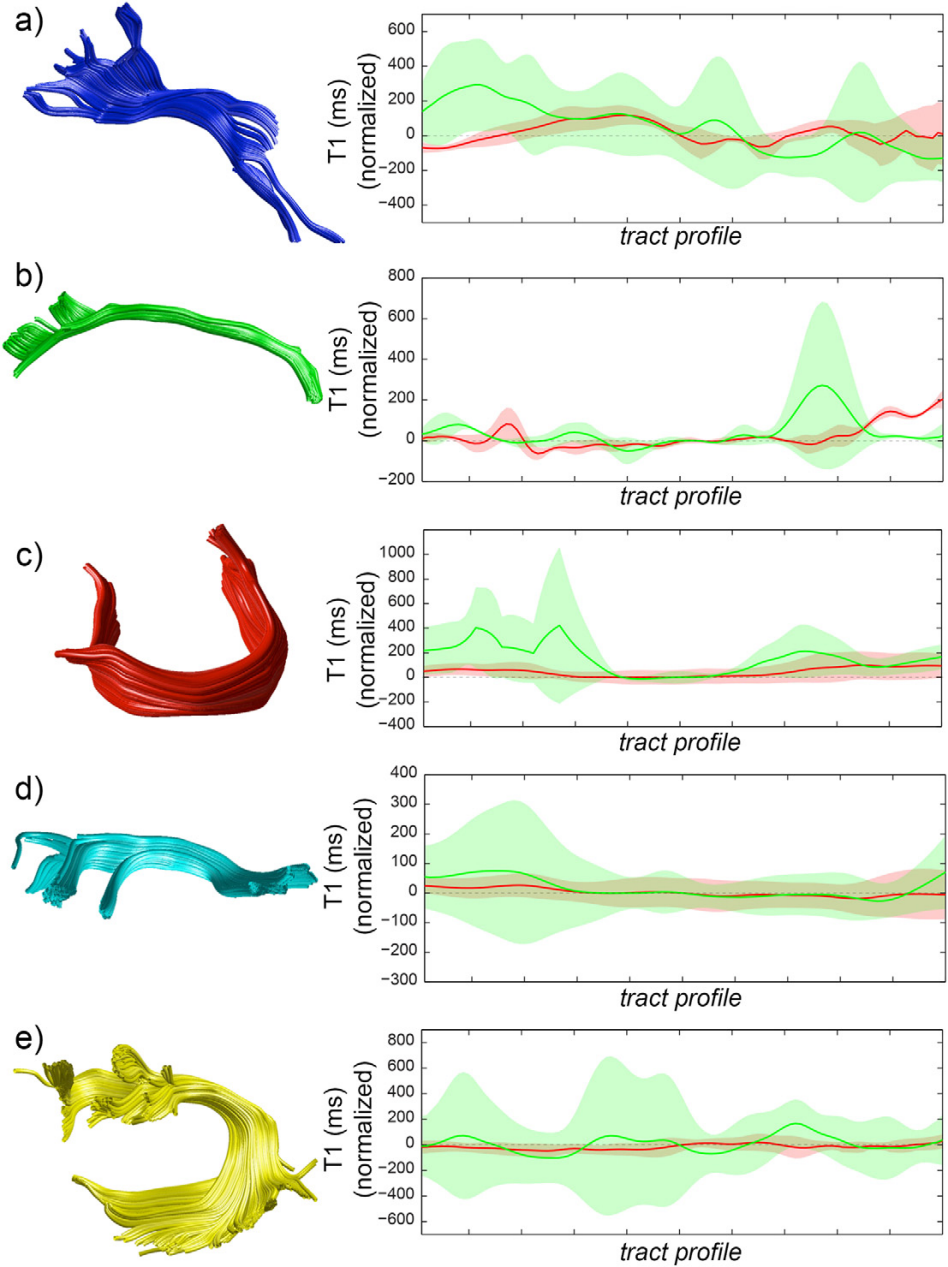
Reconstructed fibre tract of a representative subject (left) and corresponding mean tract profile. To facilitate comparison, the *T*_1_ scale is normalised to the value of *T*_1_ in the middle of the tract. Shaded areas represent the standard deviation across all the subjects (right). Profiles are reported for IR-DTI *T*_1_ (red) and for MP2RAGE *T*_1_ (green). The results are shown for five fibre tracts: the thalamic radiation (a), the cingulum (b), the corpus callosum (c), the superior longitudinal fasciculus (d) and the arcuate fasciculus (e).

Fig. 6 shows the results of the profile analysis for analysed tracts. For each tract, the mean profile (normalised to the *T*_1_ value in the middle of the tract) and the standard deviation across subjects along the tract is reported for IR-DTI *T*_1_ (red) and for conventional MP2RAGE *T*_1_ (green). The IR-DTI approach always results in lower standard deviations. In cases of known presence of multiple crossing tracts, the differences are particularly pronounced. For instance, the inter-subject variability in the centre of the CC is small for MP2RAGE *T*_1_, but increases to several times the variability of IR-DTI *T*_1_ in the more lateral parts of the CC where it crosses the CST and the SLF.

**Fig. 7:**
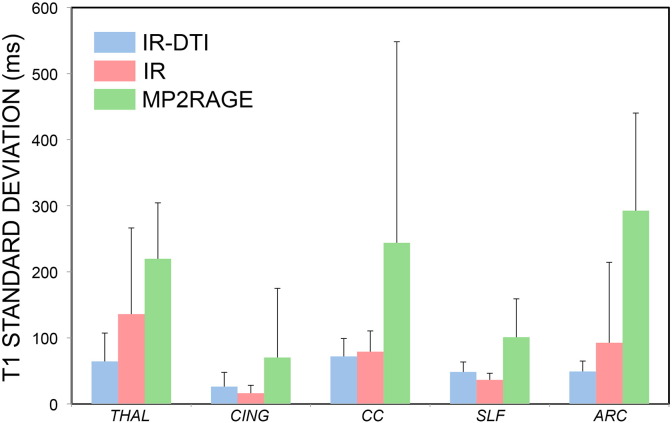
Mean and standard deviation of over-subject deviations along the five tracts reported in Fig. 6 for IR-DTI (blue), conventional IR (red) and MP2RAGE *T*_1_ tractometry (green).

Fig. 7 shows the average standard deviation along the five tracts reported in Fig. 6 for IR-DTI, conventional IR and MP2RAGE *T*_1_ tractometry. MP2RAGE *T*_1_ shows the largest variability in all tracts, followed by IR *T*_1_, while IR-DTI *T*_1_ shows the smallest variability.

## Discussion

The IR-DTI approach recently succeeded in measuring fibre-specific relaxation time *T*_1_ inside a voxel in an animal model (De Santis et al., 2015), opening a new scenario for WM investigation: the characterisation of *T*_1_ in each tract, rather than in each voxel. This approach is best suited to describe an organised structure, whose sub-units (the fibre bundles) are highly homogeneous in their composition. Here, we demonstrate the feasibility of the approach both in simulations and in-vivo in the human brain. In addition, we demonstrate the advantages of using IR-DTI over conventional, voxel-wise approach, where most of the voxels are expected to contain contributions from different bundles (Jeurissen et al., 2012).

Fig. 2 shows that IR-DTI is better than IR at separating two distinct *T*_1_s of 800 and 1000 ms in the same voxel, with the same number of measurements and the same SNR. This is likely to be due to the fact that IR-DTI is a bi-dimensional technique, because data are acquired both in the space of TI and in the space of gradient orientations (directionality). In the case where the two *T*_1_s are very close and the measures are only acquired in the TI space it is very difficult to tease apart two exponentials, unless the SNR is unrealistically high (Fig. 2, right panels). Conversely, a bi-dimensional technique that exploits the directionality of the diffusion signal to get relaxometry information along the tract successfully differentiates the two components.

IR-DTI has higher sensitivity to *T*_1_ changes than conventional IR *T*_1_, as shown in Fig. 3. Depending on which fibre is changing its relaxation properties, IR-DTI needs an effect size of only 3–6% to detect a significant change, while conventional IR needs much higher effect size (>10%). This result opens exciting scenarios for early detection of pathologies selectively affecting one fibre bundle in crossing fibre areas, like Wallerian degeneration (Pierpaoli et al., 2001), Huntington's disease (Douaud et al., 2009) and Alzheimer's disease (Douaud et al., 2011). It should be noted that IR-DTI outperforms IR especially when the fibre with the smaller volume fraction is the one affected by the *T*_1_ increase, as expected intuitively. This suggests that IR-DTI is especially advantageous in detecting the change in fibre-specific *T*_1_ for small tracts.

We were able to reproduce in the human brain the findings obtained in the animal model (De Santis et al., 2015). The difference found in the animal model between the *T*_1_ of the cingulum and that of the corpus callosum (see Fig. 9 in De Santis et al. (2015)) was also replicated in the human brain, as shown in Fig. 4. This result is consistent with the fact that the corpus callosum is more myelinated than the cingulum (see Dean et al. (2014) for developing humans and Bowley et al. (2010) for the rhesus monkey) and myelin content is inversely correlated with *T*_1_ (Lutti et al., 2013). Given the increased fibre complexity of the human brain compared to rodent, here we extended the model to include up to three fibre populations. We demonstrated using both simulations (Fig. 1) and real data (Fig. 5) that IR-DTI can successfully disentangle three compartments in each voxel. In areas likely belonging to the same fibre tract, the tract-specific *T*_1_ maps are also homogeneous, with adjacent tracts showing clearly distinct values of *T*_1_, as shown in Fig. 5. Importantly, the values recovered in the crossing area are consistent with those calculated along the tract, demonstrating the reliability of the method and also suggesting for the first time (to the best of our knowledge) that different tracts are characterised by different *T*_1_s, likely reflecting their different myelination properties.

IR-DTI measurements have less inter-subject variability along the tract, when tract-specific *T*_1_s are used, as compared to conventional IR. Notably, larger inter-subject standard deviations are observed only for the conventional IR in areas with high fibre dispersion, as seen in Fig. 6. For example, in the centre of the corpus callosum, MP2RAGE and IR-DTI provide small standard deviation. Moving away from the centre of the tract, where higher fibre dispersion and crossing with other tracts is found, MP2RAGE *T*_1_ provides increased standard deviation, while the IR-DTI *T*_1_ profile does not show any change in the standard deviation. We speculate that this is as a result of increased specificity, gained by removing the confound of fibre crossing. Fig. 7 confirms that methods based on a single *T*_1_ component result in higher variability along the tracts. In addition to the fibre crossing effect, the overall higher variability of MP2RAGE *T*_1_ may be partly explained by the fact that its much higher resolution comes with a lower SNR, especially when considered that IR-DTI is fitted over many volumes of data. Finally, it seems unlikely that going from 2 mm isotropic to 0.7 mm isotropic allows separating fibres, as in most cases the voxel will contain crossing fibres interdigitating at the level of about a hundred micron (Jbabdi et al., 2015).

With a double suppression of signal, both by the IR *T*_1_ weighting and by the diffusion weighting, SNR is of concern for IR-DTI even at high field. Here, we choose a moderate resolution (compared to resolutions achievable at high-end 3T and 7T systems) to ensure adequate SNR, obtaining SNR values between 25 and 30 across WM. However, in our simulations we have employed a conservative value of 20 for the SNR, and demonstrated that the method is able to resolve accurately up to three fibres crossing, which suggests that there is room to improve resolution.

Possible limitations for translation to the clinic are the long acquisition times that result from the elevated SAR caused by both inversion and refocusing pulses, especially at 7T. 7T helps with increased signal in the doubly attenuated IR-DTI contrast and increases the differences in *T*_1_ (Koenig et al., 1990, Rooney et al., 2007, Wright et al., 2008), and thus facilitates detection of multiple *T*_1_s, but also increases the experimental time, when TR's are increased to accommodate SAR constraints. This can be alleviated in the future, for example, by implementing state-of-the-art SAR-efficient inversion pulses (Ivanov et al., 2014).

IR-DTI can recover up to three distinct *T*_1_s inside a voxel, with minor loss in precision when the crossing angle goes from 90 to 30°, as shown in Fig. 1. It has to be noted, however, that IR-DTI relies on fibre estimates calculated using a separate HARDI acquisition, hence it will be affected by the limitations of the algorithm chosen to recover voxel-wise the fibre orientations (in this case, CSD), like the well known bias for small crossing angles (Tournier et al., 2008).

*B*_1_ inhomogeneity is challenge for 7T imaging. Using standard circularly polarized or ‘birdcage’ transmit coils yields flip angles which are up to 42% lower in the periphery of the brain compared to centrally, due to dielectric effects (Vaughan et al., 2001). To mitigate this effect, we have used dielectric pads to improve *B*_1_ homogeneity (Snaar et al., 2011, Teeuwisse et al., 2012). Improved RF pulse design, such as the use of adiabatic pulses (Tannús and Garwood, 1997), might help mitigate the problem and will be investigated in the future.

In conclusion, we demonstrate the feasibility of in-vivo IR-DTI analysis on the human brain identifying fibre-specific *T*_1_ values. IR-DTI has great potential for application in the clinic, for instance in detecting very early tract specific alterations of myelination in crossing fibre areas that might not be detected using other MRI-based approaches.

## Figures and Tables

**Table 1:** Repetition time, IR-DTI scan duration and total acquisition time (TA) for all subjects.

Subject	TR (s)	IR-DTI duration (min)	Total TA (min)
subj 1	13.5	47	62
subj 2	10.5	37	52
subj 3	14	49	64
subj 4	14	49	64
subj 5	12.5	44	59
subj 6	10	35	50
subj 7	10.5	37	52

**Table 2:** Percentile standard deviation calculated within each tract, averaged over all the subjects, for IR-DTI and conventional IR.

Tract	SD IR-DTI (%)	SD IR (%)
Thalamic radiation	8.6	12.5
Cingulum	6.6	7.6
Corpus callosum	4.8	19.7
Arcuate	4.2	12.8
SLF	3.2	5.0
